# Transcriptome Sequencing Data of Bacillus anthracis Vollum Δ*htrA* and Its Parental Strain, Isolated under Oxidative Stress

**DOI:** 10.1128/MRA.00618-20

**Published:** 2020-08-27

**Authors:** Theodor Chitlaru, Inbar Cohen-Gihon, Ofir Israeli, Uri Elia, Galia Zaide, Ma'ayan Israeli, Adi Beth-Din, Shirley Lazar, Sharon Ehrlich, Anat Zvi, Ofer Cohen

**Affiliations:** a Department of Biochemistry and Molecular Genetics, Israel Institute for Biological Research, Ness Ziona, Israel; Indiana University, Bloomington

## Abstract

The high-temperature requirement chaperone/protease (HtrA) is involved in the stress response of the anthrax-causing pathogen Bacillus anthracis. Resilience to oxidative stress is essential for the manifestation of B. anthracis pathogenicity. Here, we announce transcriptome data sets detailing global gene expression in B. anthracis wild-type and *htrA*-disrupted strains following H_2_O_2_-induced oxidative stress.

## ANNOUNCEMENT

The Gram-positive spore-forming obligate pathogen Bacillus anthracis is the etiological cause of anthrax. The lethality of B. anthracis is attributed to its exotoxins and its optimal adaptation to tolerate stress constraints encountered in the course of infection (1). Proteomic/serologic surveys of B. anthracis (1–3) showed that the secreted protease/chaperone high-temperature requirement (HtrA) (involved in protein synthesis quality control and necessary for tolerance to heat, oxidative, and other stress regimens) belongs to a class of immunogenic vaccine and disease biomarker candidates (4). Virulence-attenuating *htrA* gene disruption was implemented for the development of a live spore vaccine (5–7). Recently, we suggested that HtrA acts as both a protease/chaperone and a pleiotropic factor of gene expression (8). Here, we present transcriptome sequencing (RNA-seq) data sets describing the effect of *htrA* gene disruption on the global gene expression of B. anthracis in the presence/absence of H_2_O_2_.

B. anthracis parental strain ΔVollum (acapsular and nontoxinogenic, referred to in this report as wild type [WT]) and an *htrA*-disrupted strain, in biological triplicate or duplicate cultures (14 sets of data, as detailed in Table 1), were grown in brain heart infusion broth at 37°C to mid-log phase and split into twin cultures in the presence or absence of 3 mM H_2_O_2_. Cells were collected from the initial culture before treatment (Table 1, samples 1 and 2) and 10 min after treatment (Table 1, without H_2_O_2_, samples 3 and 5; with H_2_O_2_, samples 4 and 6). Total RNA was extracted using the RNeasy kit (Qiagen), and residual DNA was digested using RNase-free DNase (Qiagen). RNA-seq was performed in-house (IIBR, Ness Zionna, Israel). Libraries were generated using the TruSeq RNA library prep kit version 2 (Illumina), assessed for correct sizing using a high-sensitivity Bioanalyzer DNA chip (Agilent), quantified by quantitative PCR (qPCR), and normalized to 2 nM. Pooling and clustering of libraries were performed using the Illumina cBot system; 35-bp single-end sequencing was performed on the Illumina Genome Analyzer IIx system with TruSeq sequencing-by-synthesis (SBS) kit version 2 reagents. FastQC (https://www.bioinformatics.babraham.ac.uk/projects/fastqc) was used for quality control of the data. Reads were mapped to the B. anthracis Ames Ancestor reference genome (GenBank accession number NC_007530) using Novoalign, version 3.02.07. The HTSeq software (9), version 0.6, was used to quantify the number of reads mapped to each gene. Sequencing yielded 4.3 million to 11.9 million reads (Table 1) with a mapping percentage that ranged from 89.3% to 99.5%. An analysis of differentially expressed genes under various conditions was performed using the R package DESeq, version 1.16.0 (10). All analytical software programs were used at their respective default settings.

**TABLE 1:** Summary of transcriptome samples in this study^*a*^

Sample^*b*^	Significance	No. of reads	% mapped reads^*c*^	SRA accession no.	GEO accession no.
1a	WT before treatment with H_2_O_2_	8,753,632	99.3	SAMN15018196	GSM4568574
1b	WT before treatment with H_2_O_2_	4,341,085	99.5	SAMN15018194	GSM4568575
2a	Δ*htrA* before treatment with H_2_O_2_	11,953,954	99.2	SAMN15018192	GSM4568576
2b	Δ*htrA* before treatment with H_2_O_2_	8,344,835	99.2	SAMN15018191	GSM4568577
3a	WT in the absence of H_2_O_2_ (WT noninduced control)	7,481,324	92.5	SAMN15018189	GSM4568578
3b	WT in the absence of H_2_O_2_ (WT noninduced control)	8,944,123	97.2	SAMN15018187	GSM4568579
4a	WT 10 min after treatment with 3 mM H_2_O_2_	7,716,866	93.3	SAMN15018185	GSM4568580
4b	WT 10 min after treatment with 3 mM H_2_O_2_	8,398,131	99.2	SAMN15018181	GSM4568581
4c	WT 10 min after treatment with 3 mM H_2_O_2_	9,706,981	92.0	SAMN15018200	GSM4568582
5a	Δ*htrA* in the absence of H_2_O_2_ (Δ*htrA* noninduced control)	8,799,194	95.8	SAMN15018198	GSM4568583
5b	Δ*htrA* in the absence of H_2_O_2_ (Δ*htrA* noninduced control)	9,429,758	96.1	SAMN15018203	GSM4568584
6a	Δ*htrA* 10 min after treatment with 3 mM H_2_O_2_	7,632,366	89.3	SAMN15018205	GSM4568585
6b	Δ*htrA* 10 min after treatment with 3 mM H_2_O_2_	6,270,715	91.2	SAMN15018202	GSM4568586
6c	Δ*htrA* 10 min after treatment with 3 mM H_2_O_2_	8,221,717	94.3	SAMN15018201	GSM4568587

a The SRA BioProject number is PRJNA635127. The GEO title of the project is “Transcriptome RNA Sequencing Data Sets of Bacillus anthracis Vollum Δ*htrA* and Parental Isogenic Wild-Type Strains under Oxidative Stress Conditions.”

b Biologically duplicated samples are labeled as a and b, and triplicated biological samples as a, b, and c.

c Percentage of reads mapped to the reference genome of B. anthracis Ames Ancestor (NCBI accession number NC_007530). The GEO accession number of the transcriptome series is GSE151208.

The analysis revealed the following categories of H_2_O_2_-modulated genes: (i) induced upon treatment in both strains (792 genes), (ii) repressed in both strains (868 genes), (iii) uniquely upregulated in the WT strain (271 genes), (iv) uniquely downregulated in the WT strain (221), (v) uniquely upregulated in the mutant (330 genes), and (vi) uniquely downregulated in the mutant (648 genes). Further inspection of these classes of genes will enable a better understanding of the response of B. anthracis to oxidative stress in general and the regulatory role of the protein HtrA in particular. Furthermore, this database may facilitate identification of proteins for the future development of countermeasures against B. anthracis.

### Data availability.

The transcriptomic data have been deposited in the NCBI database, and their SRA and GEO accession numbers are provided in Table 1.
